# Supplementary material for: Towards clinical applicability of fMRI via systematic filtering
Source: PLoS One. 2025 May 12;20(5):e0321088. doi: 10.1371/journal.pone.0321088 (PMC12068634; doi:10.1371/journal.pone.0321088)
Supplement: S1 Code — (ZIP) [file pone.0321088.s001.zip › PLOS_DATA_CODE/Welcome to the code.docx]

Welcome to the code!

Inside this folder, you’ll find code that we crafted for our own purposes, so it hasn’t been tailored to run with your specific data. But fear not—if you’re looking to reproduce our results, simply utilize the “find_filter.m” function located in the Code folder to discover the optimal SG filters.

Curious about the effects of this filter on other data? Dive into the same folder and check out “test_filter.m,” where you can see the filter in action on a different dataset. For those eager to explore our simulations, head over to the Simulate folder and find “simulate_filt.m.”

Just a friendly reminder: don’t forget to rename the path where you have installed the folder in the respective MATLAB scripts. Also, a quick note—some of this code was generously borrowed from programmers of greater talent. The SPM functions were crafted by the skilled John Ashburner, and while “extract_rt_rel_response_filter.m” features our input, it also draws inspiration from an anonymous author. While the code is entirely of our own making, the code comments were partly improved by ChatGPT-4.

Like any code, it has been made by humans and may contain errors. We would appreciate it if you could communicate any errors you find.

Please do not use this code for medical applications, as it has no legal status for such use. Please use FDA-approved software.

Cheers,

Jan Koten
