# Supplementary material for: Towards clinical applicability of fMRI via systematic filtering
Source: PLoS One. 2025 May 12;20(5):e0321088. doi: 10.1371/journal.pone.0321088 (PMC12068634; doi:10.1371/journal.pone.0321088)
Supplement: S2 File — (PDF) [file pone.0321088.s002.pdf]

Supporting Information

Towards clinical applicability of fMRI via systematic filtering

S1 Text. Sample, task and methods

Sample

We established a sample (n=67) that is representative for the Austrian population in the age range 18-75. The sample consisted of 39 females and 28 males. No individuals were excluded on the grounds of race, education or head motion parameters. However, effects of motion on imaging quality are reported under the heading motion correction. The histogram depicted below gives the age distribution of the sample.

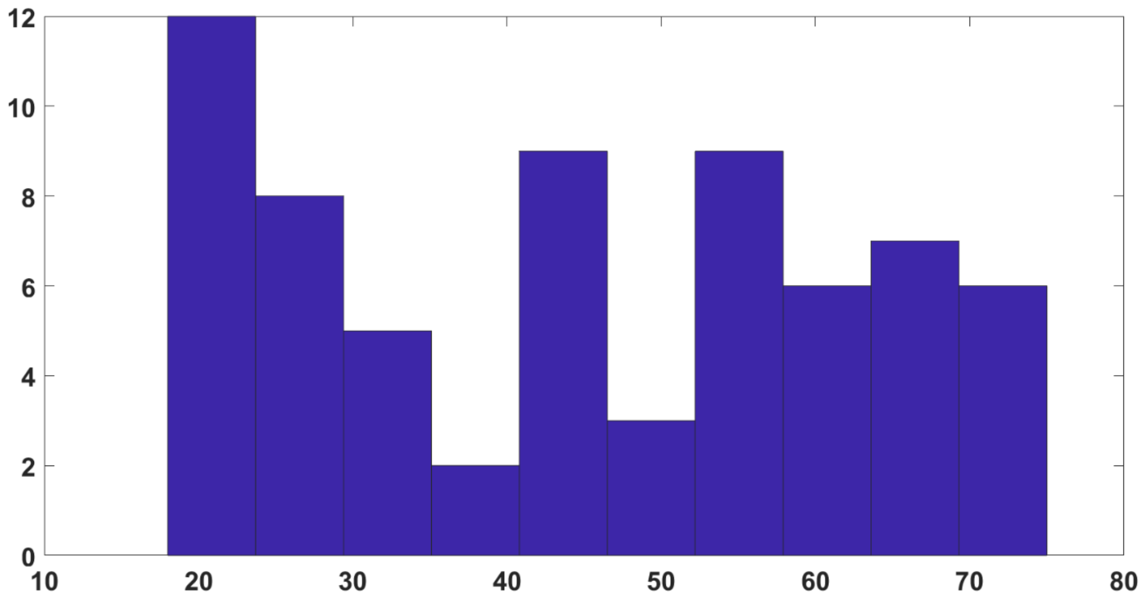

S1 Text Figure 1: This histogram reports the age distribution of the sample.

Working memory tasks

Prior to scanning participants were trained for the tasks under study until they performed confidently. Individuals had to retain two letters or two spatial positions in working memory while performing a number stroop task. During the encoding phase a set of two letters or two spatial positions was memorized; subsequently individuals had to perform a number Stroop task; finally, individuals had to press a button with the right index finger when a newly presented item was part of the previously presented item set. The left index finger was used in case of non-correspondence. The number stroop tasks consisted of two simultaneously presented Arabic numbers that were presented in two physical sizes. Individuals had to identify the larger numerosity and use the right finger when the larger number was found on the right side of the screen and vice versa. In the congruent condition the larger numerosity corresponded with the larger physical size of the Arabic digit while the opposite was true for the Stroop condition. The verbal and spatial items presented during the encoding phase were spatially randomized and positioned within the cells of an invisible 4*4 matrix. After the last item of the behavioural experiment a jittered resting baseline condition time was presented.

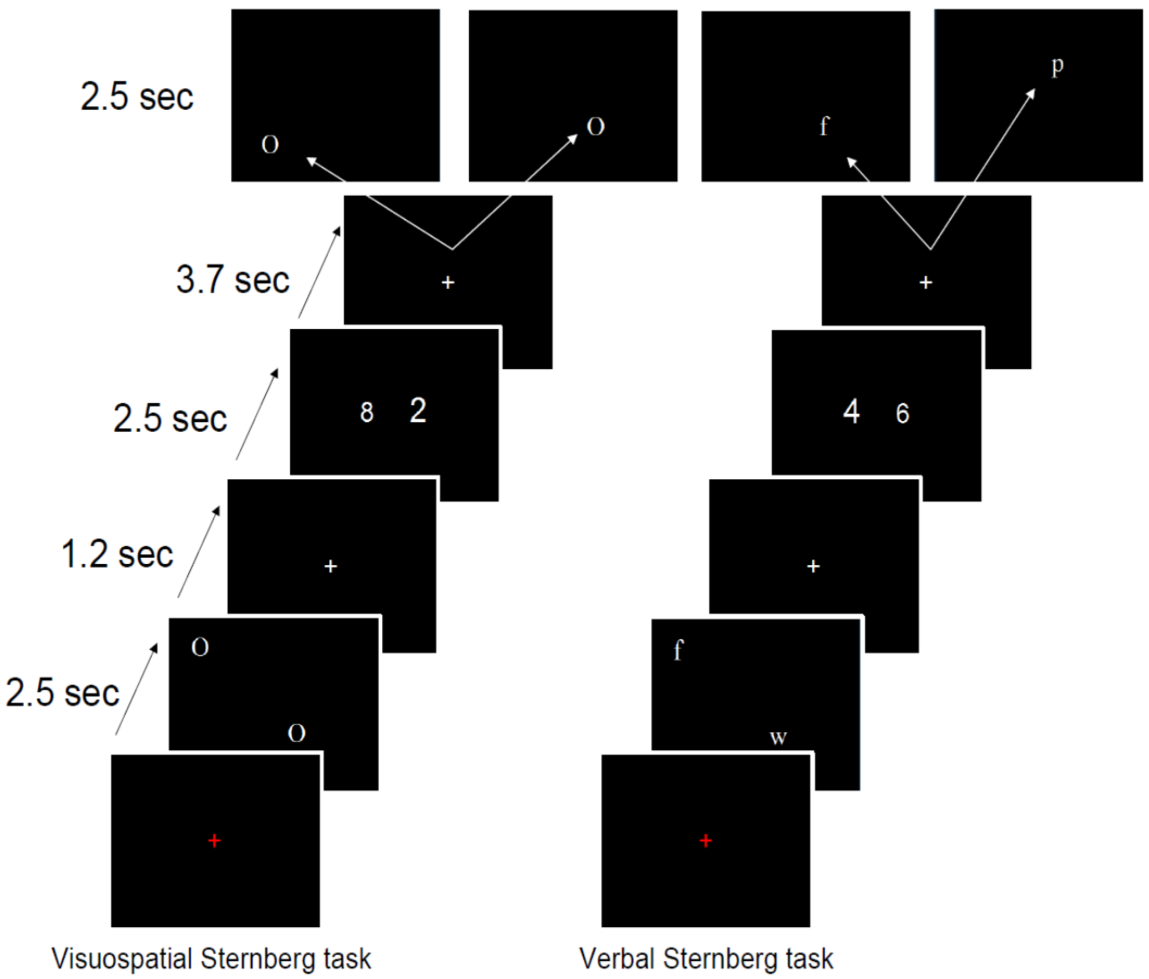

S1 Text Figure 2: This figure illustrates details of the spatial and verbal working memory tasks used for the optimization and validation experiment respectively. Stimulus duration is depicted on the left side. The resting condition indicated by a red fixation cross was jittered to a duration between 11.16 sec. and 14.88 sec. Individuals memorized the spatial position of two "O's" and had subsequently to identify the larger numerosity of two Arabic numbers that differed in physical size by pressing a button with the left or right index finger. Finally, participants had to verify whether the spatial position of a newly presented "O" was in agreement with the previously memorized "O" set. If this was the case, participants pressed the right button, if not, the left one. Right side, the verbal memory task had the same structure. Participants verified the identity of the letter (same/different) by pressing a button with the left or right index finger.

Co-registration and segmentation and motion correction

Prior to registration slice time correction and motion correction of functional data was performed. We estimated head motion as the largest possible Euclidian distance in mm between points in space. Contrary to standard practice we did not exclude individuals with larger head motion from the sample. We did this for two reasons. First, we used a stratified sample that represents the Austrian populations including all age (>18) and education classes. Excluding individuals with larger head motion would have led to a sample were some classes of individuals would have been omitted.
Second, the main of this study is to improve reliability trough SG filters. We believe that individuals with larger head motion are useful to demonstrate the favourable properties of these filters. We nonetheless want to give the reader and idea of how head motion may affect test-retest reliability on the within subject within ROI level. For this analysis we sorted all 67 individuals according to their head motion parameters. In first step we selected six individuals that exhibited the smallest head motion and estimated the grand mean within subject time course reliability from this subsample. In a next step the sliding window omitted the subject with the smallest head motion from the list and added the subject that was seventh on the head motion list. This procedure was repeated until the subsample with the largest head motions was studied for the distinct pre-processing methods.

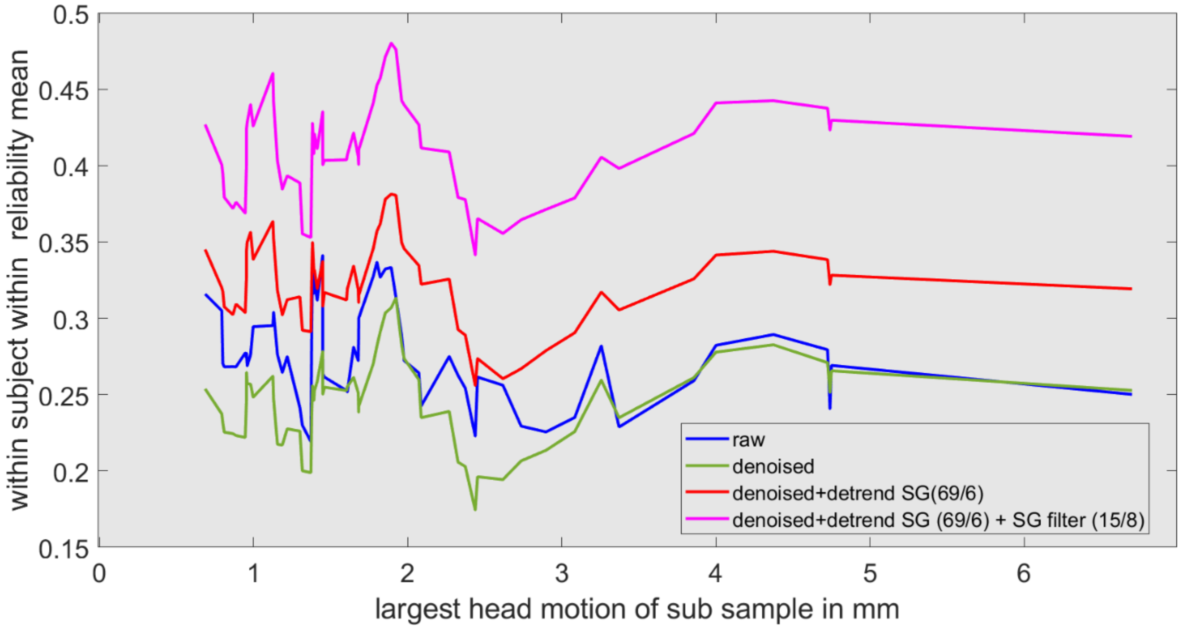

S1 Text Figure 3: This diagram reflects mean test-retest reliability as function of head motion expressed in mm. The approach was based on a sliding window that included a subs sample of 6 individuals. Mark that only six individuals exhibit head motions > 4 mm.

The Free surfer (FS) pipeline consists of two sub pipelines one in 3D volume space and one in 2D mesh surface space. In this study the 3D volume pipeline was used to co-registrate the functional images obtained in the test and retest run with the structural image obtained in the test run. Moreover, the nuisance time courses of the white matter, ventricles and head motion were extracted using the 3 D pipeline. The 2D pipeline was used to align the grey matter time courses to FS average space with high accuracy.

Alignment

Here we used spherical alignment as provided by the free surfer package. The main advantage of this approach is that it is still one of the more accurate registration methods available. This method is particularly suitable for aging studies where large individual differences in anatomy are to be expected. A further advantage of spherical alignment is that it creates high anatomical correspondence between individuals without spatial smoothing of the functional data. 3D spatial smoothing is a weighted averaging procedure which means that irrelevant time courses belonging to the white matter and dura mater etc. are averaged with the grey matter time course. Moreover, spatial smoothing might link functional distinct areas located on opposing sides of a sulcus and possibly constitute to the creation of functional connectivities that are not present in the true world. The latter may corrupt the true connectivity estimates between brain nodes. In short, the functional data of the test and retest run were aligned with the 3D structural anatomy of the test run using commands form FsFast. The quality of the alignment procedure was visually inspected in all cases. No mismatches were observed. Next the structural scans of the test run were automatically segmented into white and grey matter mass using the standard “recon all” pipeline of the FreeSurfer package. The resulting white and grey mater segmentations were visually inspected and hand corrections were made when needed. The corrected volumes were re-entered into the FreeSurfer pipeline such that accurate spherical representations of the cortex in native space were obtained. The corrected spheres were aligned with the free surfer FS average brain using spherical alignment approaches. The resulting sphere reg files were used in a later stadium to align the functional data of the test and retest run to the common FS average mesh. Next the 3D white matter volumes were eroded with two iterations. This procedure avoids that grey matter and white matter time courses might show spatial overlap. Finally, the volumes of the ventricles were defined and used to obtain nuisance time courses.

Patch of interest creation and grey matter time course extraction

In this study working memory experiments were used to investigate the functional connectivity of frontal parietal systems. We did not create our patches of interest on the basis of activation maxima potentially present in our own sample because this might cause circularity. Instead, we selected 34 MNI coordinates of interest from a recently published meta-analysis that focused on the executive aspects of working memory (Nee et al). These coordinates were brought into FS average mesh space using the procedure described at the FreeSurfer home page (surfer.nmr.mgh.harvard.edu/fswiki/VolumeRoiCorticalThickness). In short, the 3D coordinates of the ALE meta-analysis were brought into 3D FS average space using fslregister command. Next the co registered coordinates were projected onto the FSaverage mesh using the FreeSurfer mri_vol2surf command. The relevant mesh elements were brought into MATLAB® format for further preprocessing using the FreeSurfer/MATLAB® command mri_read. We draw a circle with a diameter of 8mm around the relevant mesh element using the MATLAB® surfstat command SurfStatROI (http://www.stat.uchicago.edu/faculty/InMemoriam/worsley/research/surfstat/index.htm). The resulting vertices were aggregated into a patch of interest (POI) that was used to extract the time courses from the individuals under study. All the mesh time courses of a specific POI were averaged and used to estimate the connectivity among nodes as well the test-retest reliability.

S2 Text. Simultaneous filter and nuisance regression

Previous research on resting-state data suggested that noise is reintroduced into the time-courses of interest when filters are applied after denoising (Hallquist et al. 2013). It is suggested that filters should be applied within the GLM framework. Hence, our pipeline can be criticized because we indeed optimized and validated cleaning filters after denoising and detrending data within a GLM framework. However, bandpass filters as applied in resting-state research are not suitable for cognitive research because they may corrupt the cognitive signal of interest. We investigated the behavior of the time course when cleaning filters were incorporated with the nuisance time courses in the GLM: We observed that the effects were different for the distinct filter types under study. RMSE values reported in Table S4 suggest that the autocorrelations of the SPM pipeline dropped when cleaning filters were integrated into the GLM. The better temporal resolution led to a slight loss of true detectable connectivity (Table S4). Altogether, SPM filters may improve when they are integrated into the GLM although it should be stated that the autocorrelations at lag 2-4 aver alarmingly high. Thus, autocorrelations and relative overestimation estimates of SPM cleaning filtered data remain problematic regardless of the pipeline employed. For SG filters, results were heterogeneous. We again optimized a filter in the verbal working memory task using brute force methods. As discussed, all nuisance time courses and filters were entered into the GLM at once. This led to an optimal SG cleaning filter with a window size of 105 and a polynomial order of 35. Validation of the filter in spatial working memory data led to a boost in true detectable connectivity. Nonintegrated SG filters exhibit an average true detectable connectivity of 0.43 while integrated filters exhibit a true detectable connectivity of 0.48 (Table S5). In addition, no less than 94% of individuals exhibit a mean node reliability of 0.4 when integrated cleaning filters were used while this number dropped to 70% when non-integrated cleaning filters were used. Superficially, integrated SG cleaning filters seem to perform better. But the price for higher reliability is a substantial increase in temporal autocorrelation (Table S4). In this context, RMSE of non-integrated filters was roughly 0.05 while integrated approaches exhibited a RMSE of 0.15. The corrupting effects of the integrated approach on the time-course of interest can be shown on the basis of the power spectra (Figure S12). With regard to the use of integrated filters, observations made for resting-state experiments employing FFT based filters do not necessarily generalize to working state experiments employing SG filters.

S3 Text. Monte Carlo Simulation

Research has demonstrated that the implementation of cleaning filters can elevate autocorrelations of time courses, ultimately increasing the deviation of connectivity distributions. Consequently, it is crucial to investigate whether the application of optimized SG filters, may bias time course reproducibility, which can compromise detectable connectivity. To address this concern, we conducted a post hoc Monte Carlo simulation to assess the impact of a specific SG and gaussian cleaning filter on the consistency of time course reproducibility as a function of time course autocorrelatedness. Due to the computational expense of the simulation, we focused only on the SG (15/8) filter and the Gaussian filter with a duration of 2.48 seconds. We assumed that time courses adhered to an AR(1) lag 1 model. For the entire AR(1) correlation range of 0.01-0.99 and in increments of 0.01, we assessed the effect of the cleaning filters on time course reproducibility, executing one million simulations for each iteration step and a total of 99 million iterations. We further considered that the time courses could follow a random normal distribution with stationary behavior. We generated two discrete time courses with an equal and known autocorrelation pattern and applied the simulated time courses to the cleaning filters. We compared the autocorrelation and test-retest reliability of the two simulated time courses pre-and post-cleaning filtering and estimated the standard deviation of their test-retest correlation distributions. Our simulation utilized the commented script simulate_filt.m employing the function createAutoCor.m, which is downloadable from the link provided at the conclusion of the study. Application of an SG (15/8) cleaning filter to denoised and SG detrended data boosted the autocorrelation from 0.51 to 0.79 (see Table 1). Application of an SG (15/8) filter to simulated time courses with an autocorrelation behavior of 0.51 lead to an autocorrelation of 0.81 (Figure S13 top). In analogy, the use of a Gaussian filter on simulated time courses with an autocorrelation behavior of 0.54 resulted in an autocorrelation of 0.95, while an autocorrelation of 0.93 was observed for real data. This finding suggests that simulations based on an AR(1) model could be an appropriate tool to represent real-world data accurately. The graph depicted in Figure S13 (middle) demonstrated that the standard deviations of time course reliability estimates increased as a function of time course autocorrelatedness. The most significant filter bias arose when time courses showed low autocorrelations. In contrast, standard deviations of time course reliability estimates obtained from highly autocorrelated time courses were not substantially affected by cleaning filters. The standard deviation of the simulated reproducibility distribution indicated that time courses with an autocorrelation of 0.51 had a value of 0.059 for unfiltered data, 0.076 for SG filtered (15/8) data, and 0.11 for a standard Gaussian filter (Figure S13 middle). Similarly, an autocorrelation level of 0.54 standard deviations of 0.062, 0.076 and 0.11 were observed for nonfiltered, SG filtered and Gaussian filtered data respectively (Figure S13 middle). The difference in standard deviation between SG filtered and unfiltered data equaled SD=0.016, with a notably smaller bias induced by the SG filter when compared to the Gaussian filter. This difference is particularly visible in the histograms that report the distributions of time course reliability for unfiltered, SG filtered, and Gaussian filtered data (Figure S13 bottom).

S1 Figure. Task related signal average

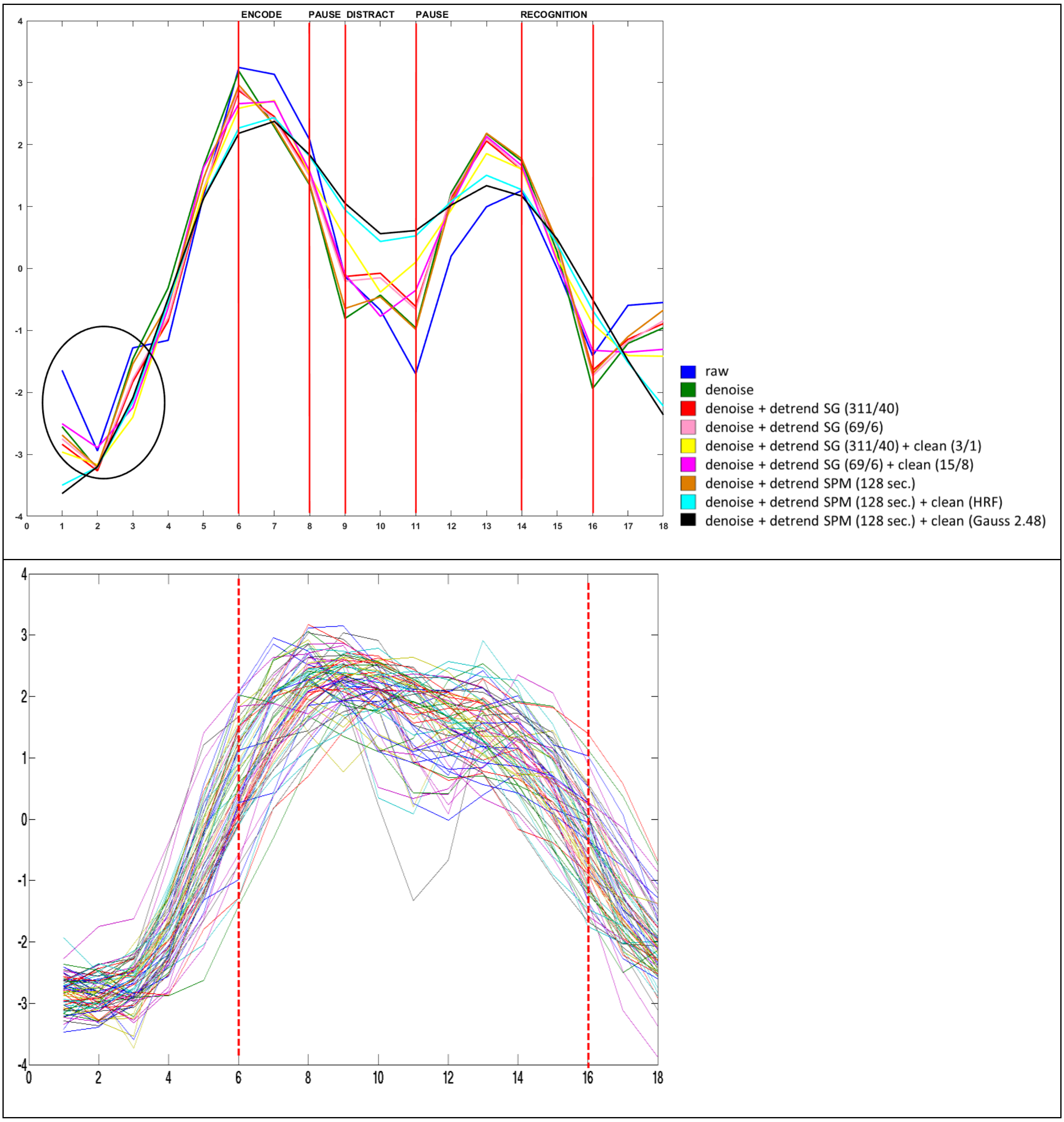

S1 Figure: Relates the cognitive events to the empirical BOLD response. The cognitive events are entitled in the upper most part of the figure.

Top: The effects of pre-processing on the event-related average of a random subject. The pipelines in question are color coded according to the legend on the right side of the figure.

Bottom: Event-related averages of all participants treated with an SG filter based pre-processing pipeline including detrending SG (311/40) and cleaning filtering SG (3/1). Time-courses were extracted from the left DLPFC.

S2 Figure. Effect of detrending

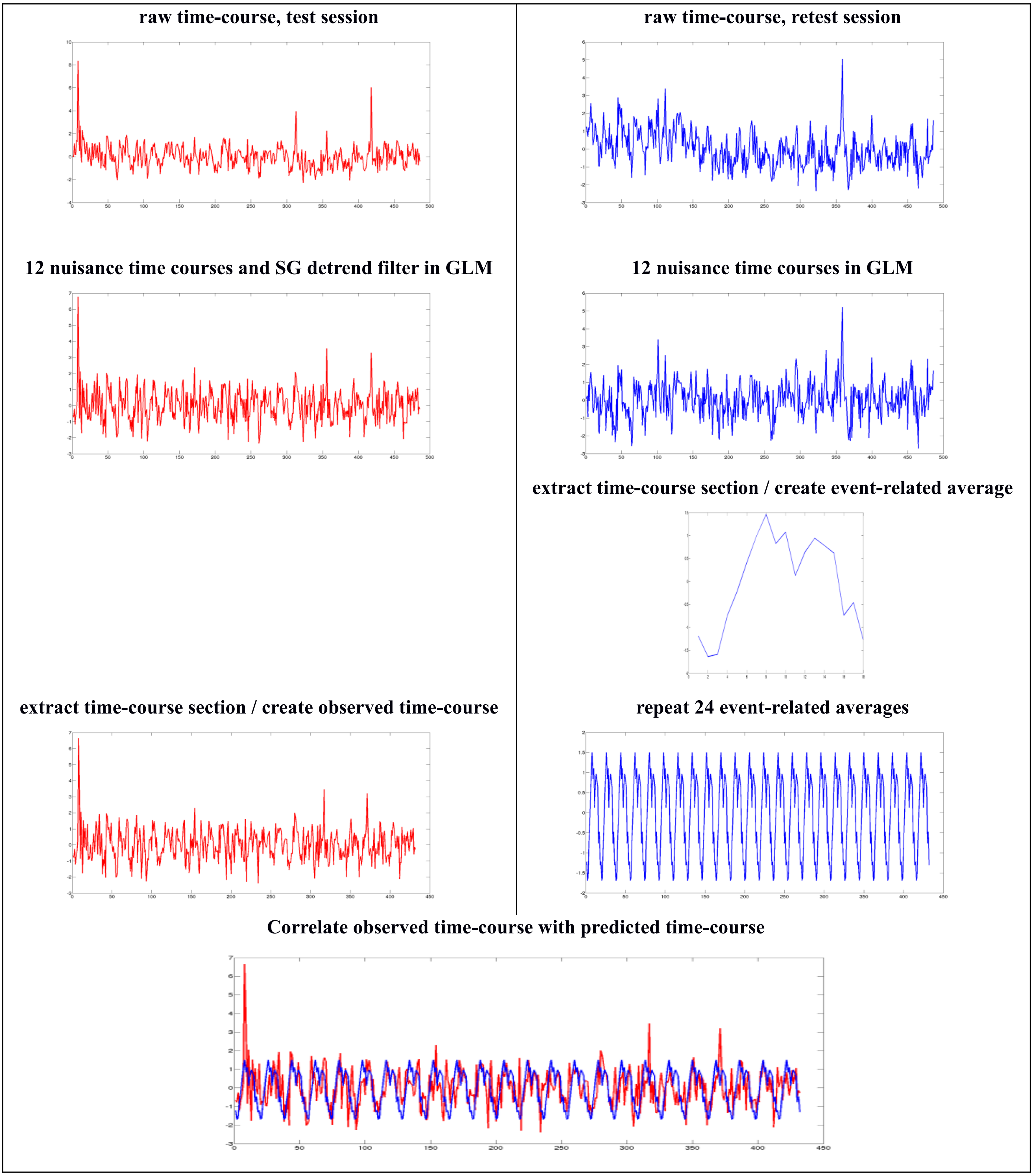

S2 Figure: Effects of pre-processing on the time-course as executed in the first phase of the optimization experiment that aimed at finding optimal detrending parameters. The left side of the figure shows how the observed time-course was treated (red) while the right side of the figure shows how the empirical predictor time course was obtained (blue).

S3 Figure. Effect of cleaning

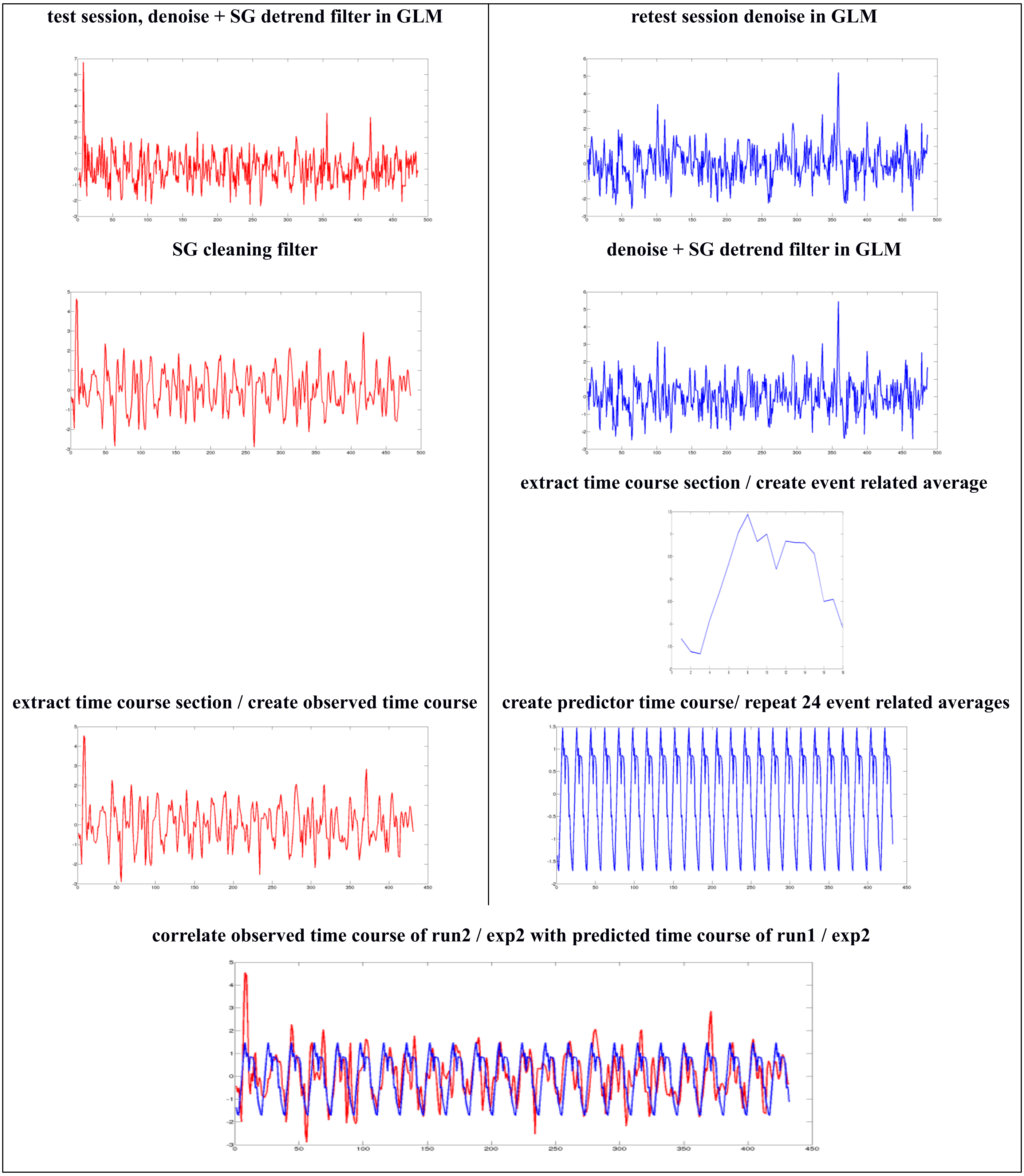

S3 Figure: Effects of pre-processing on the time-course as executed in the second phase of the optimization experiment that aimed at finding the optimal cleaning filter. The left side of the figure shows how the observed time-course was treated (red) while the right side of the figure shows how the empirical predictor function was obtained (blue).

S4 Figure. Correlation between a predictor and observed time course for given SG filter

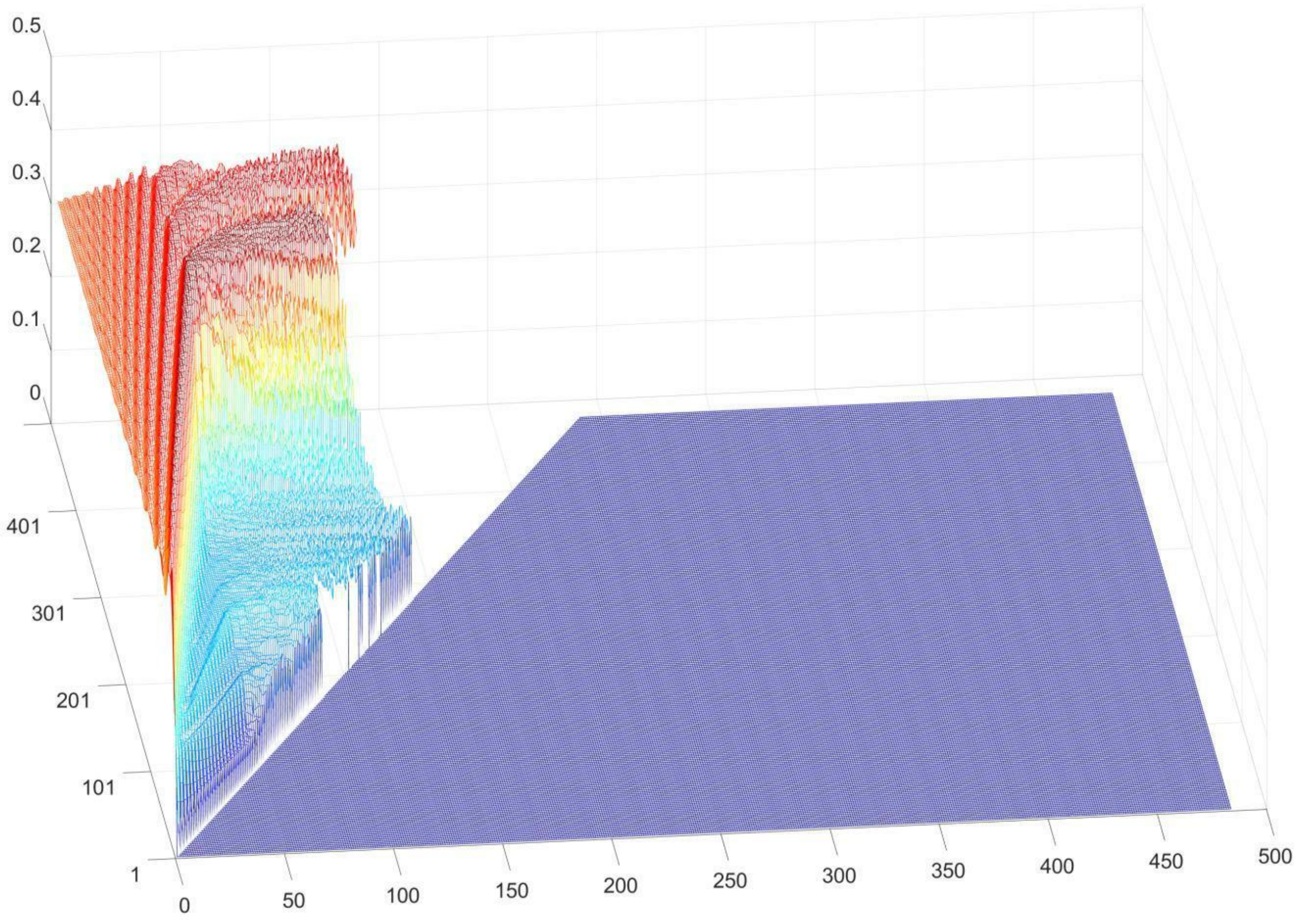

S4 Figure: This figure depicts the average correlation between a predictor and observed time course for given SG filter as obtained from 34nodes*67subjects*2runs. The z axis represents the height of the correlation the left axis the size of the window (only uneven numbers) the right axis represents polynomial order. Mark that wrong filter parameters result in zero or correlations or correlations that cannot be estimated. Filters with polynomials larger than 42 exhibit irrational behaviour.

S5 Figure. Correlation between a predictor and observed time course for a given SG filter

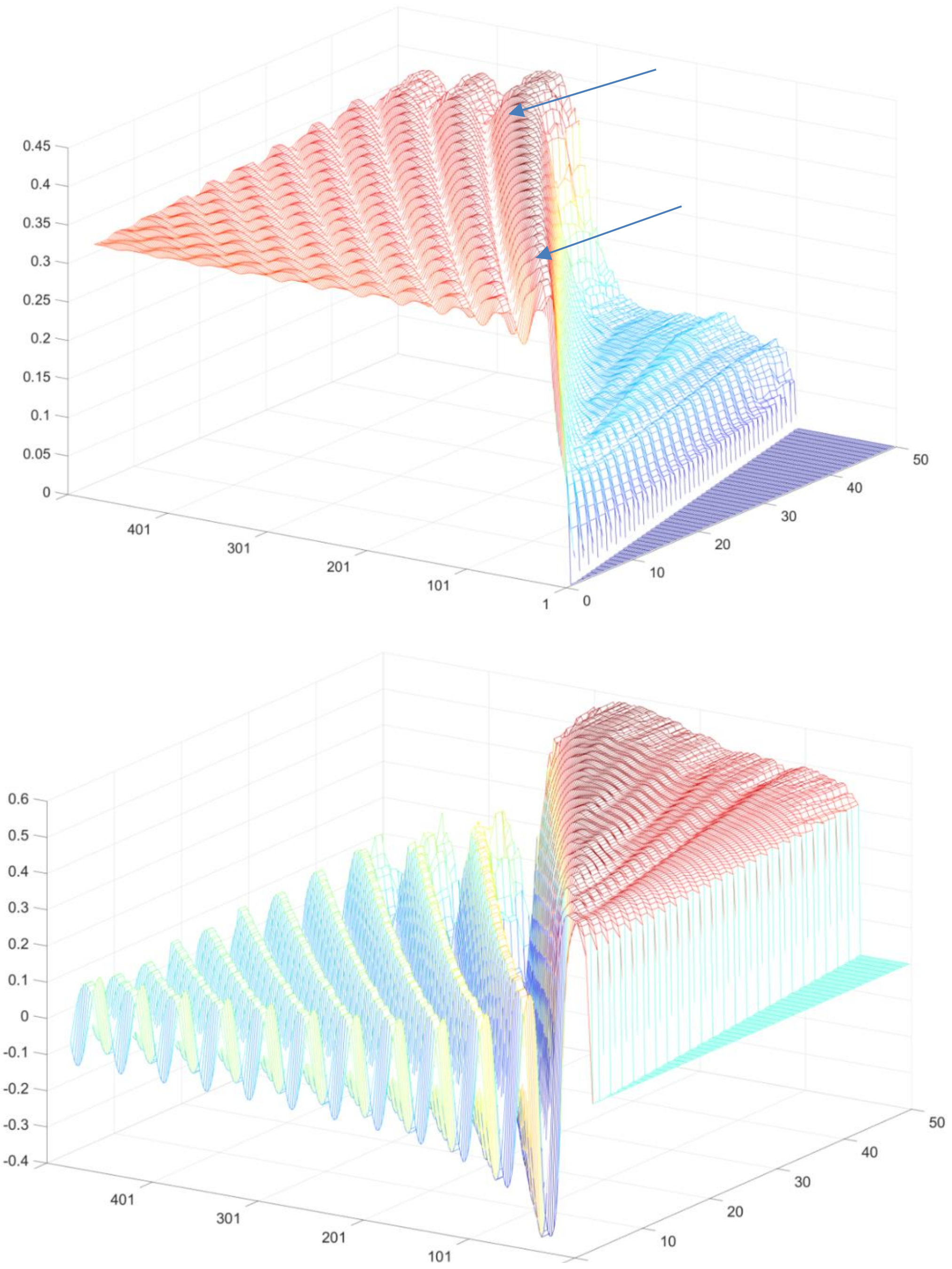

S5 Figure: Average correlation between a predictor and observed time-course for a given SG filter as obtained from 34nodes*67participants*2runs. The z-axis represents the height of the correlation, the left axis, the size of the window (only odd numbers), the right axis represents the polynomial order. Inadequate filter parameters exhibit very small or negative correlations. The arrows indicate a family of SG filters with good properties. Top: this graph shows results for a SG detrending filter that was developed in concert with denoising. Bottom: this graph shows the results for a high frequency filter obtained after denoising and SG detrending (311/40).

S6 Figure. Effect of filter on autocorrelation

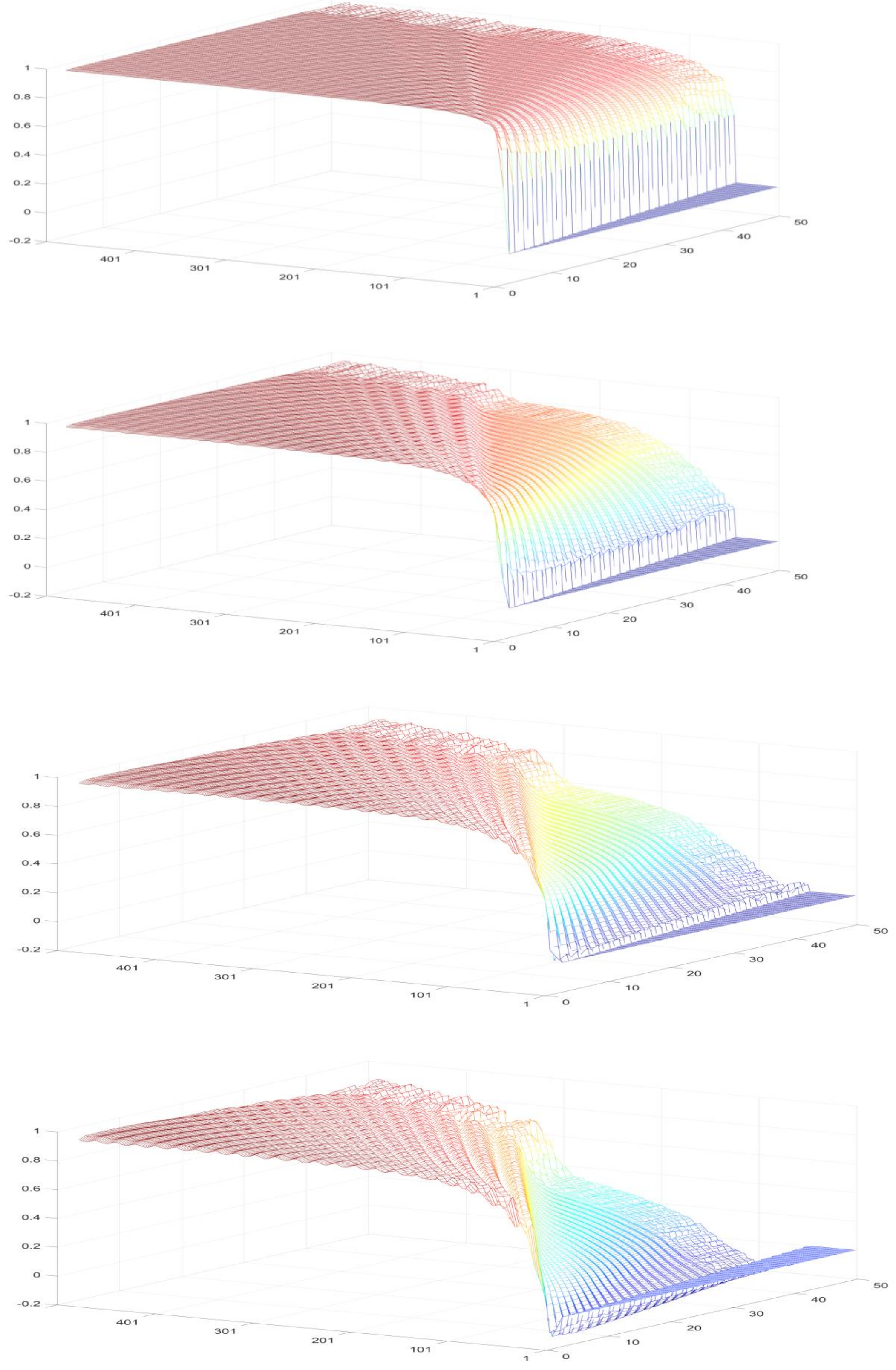

S6 Figure: This figure depicts the average autocorrelation of observed time-courses for a given SG cleaning filter obtained from 34 nodes*67participants*2runs. From top to bottom the lag 1 to lag 4 autocorrelations are represented. The z-axis represents the height of the autocorrelation the left axis the size of the window (only uneven numbers) the right axis represents polynomial order. Inadequate filters yield autocorrelations that approach 1.

S7 Figure. RMSE optimization

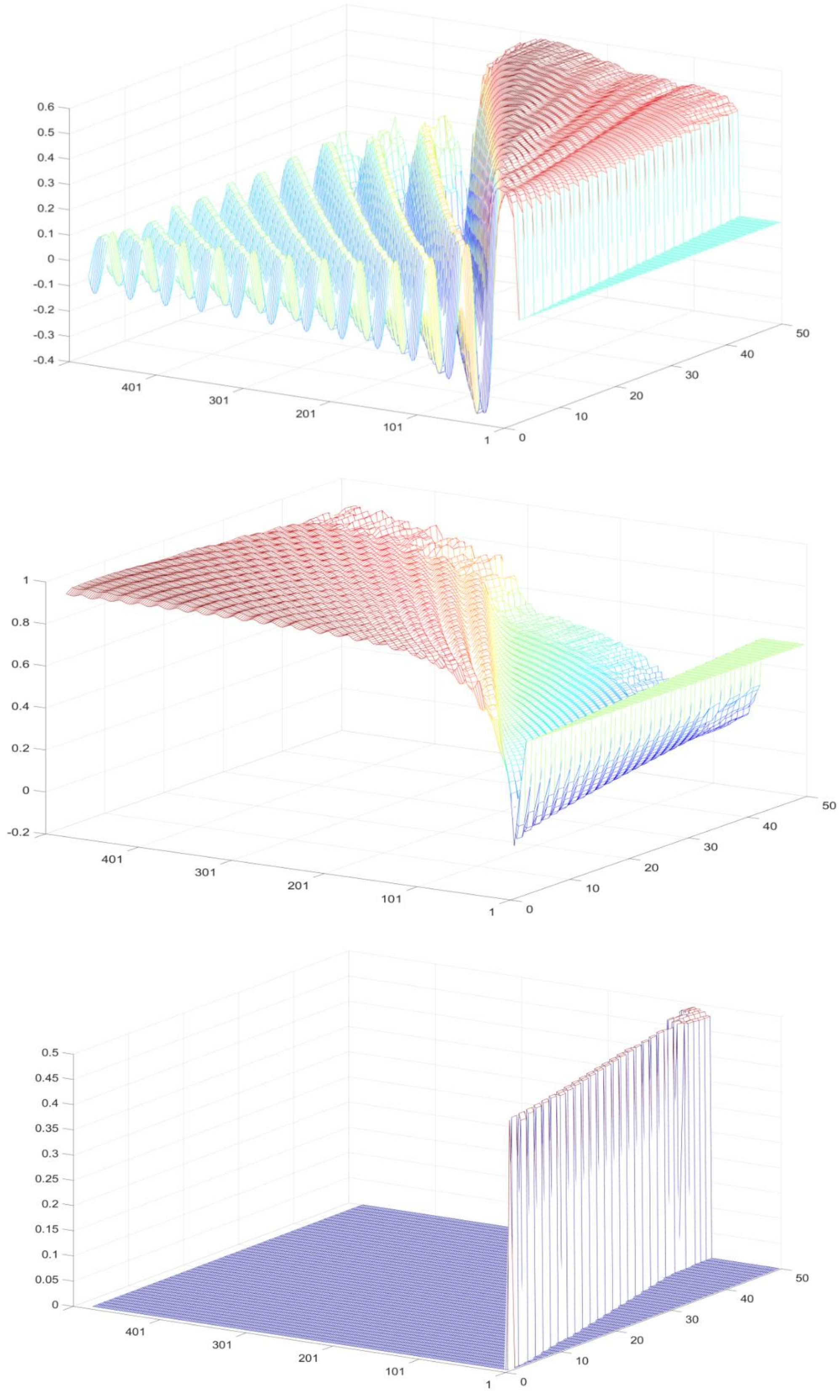

S7 Figure: Depicts the effect of SG detrend filter parameters on the correlation between predicted and observed time-courses; and the effect of SG parameters on RMSE obtained from predicted and observed autocorrelations. All data were obtained from 34 nodes *67participants*2runs. Predicted and observed time-courses underwent optimal SG detrending (311/40). Top: The z-axis represents correlation height, the left axis the size of the window (only uneven numbers), the right axis represents polynomial order. Middle: The z-axis represents RMSE the left axis the size of the window (only uneven numbers) the right axis represents polynomial order. Bottom: This figure shows what remains of the height of the correlation between predicted and observed data (top panel) when only data are selected with RMSE<0.1 (middle panel).

S8 Figure. Effect of denoising and detrending

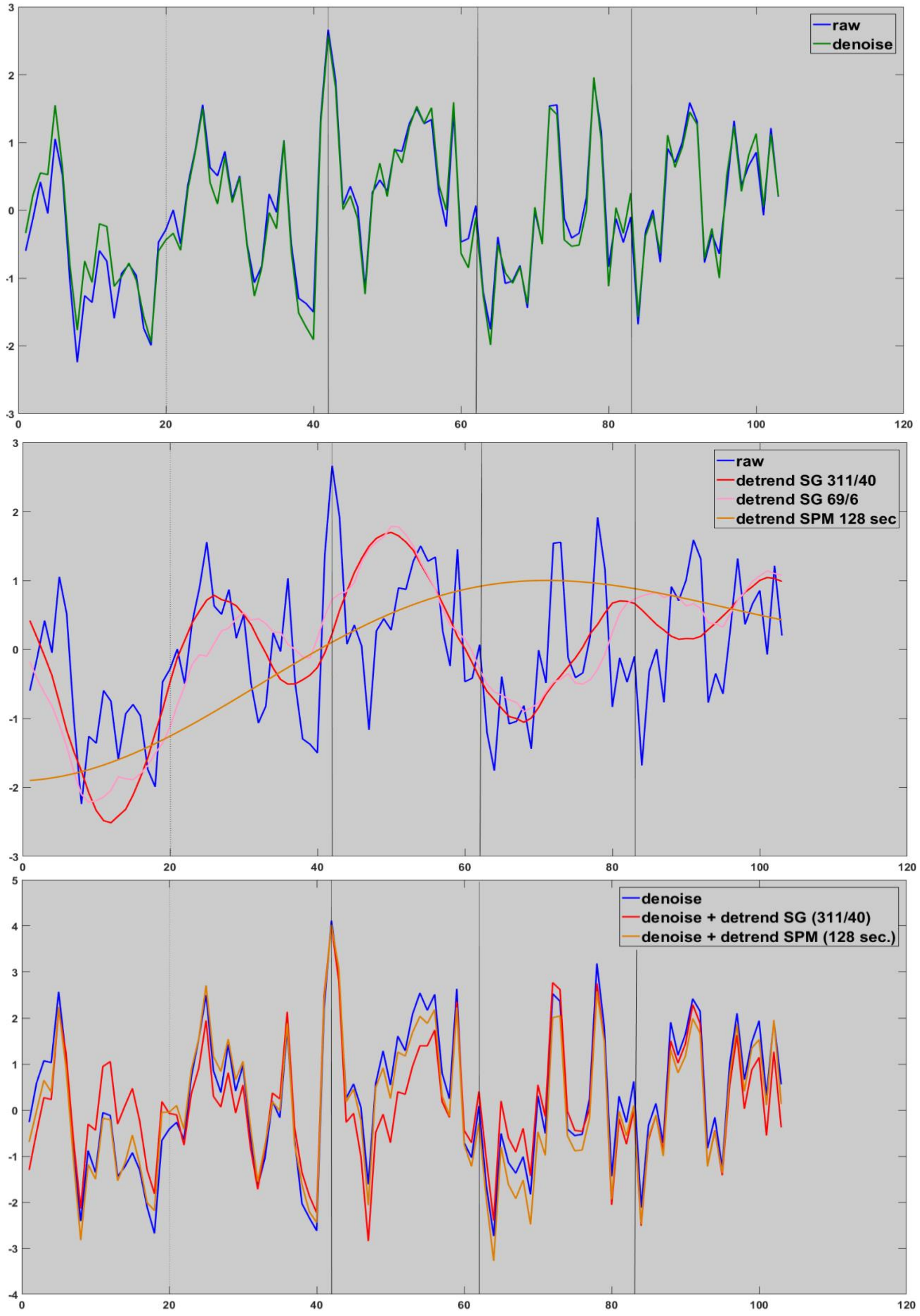

S8 Figure: Effect of denoising and detrending on a smaller fraction of the time-course. Top: effect of denoising within GLM. Middle: conventional SPM filter detects very slow fluctuations while SG filters trace oscillations in frequencies between 0 Hz and 0.04Hz Hz. Bottom: The effects of denoising and detrending executed within a GLM framework. The color in the legends depicted in the right upper corner refer to the pipeline in use.

S9 Figure. SG Reliability improvements

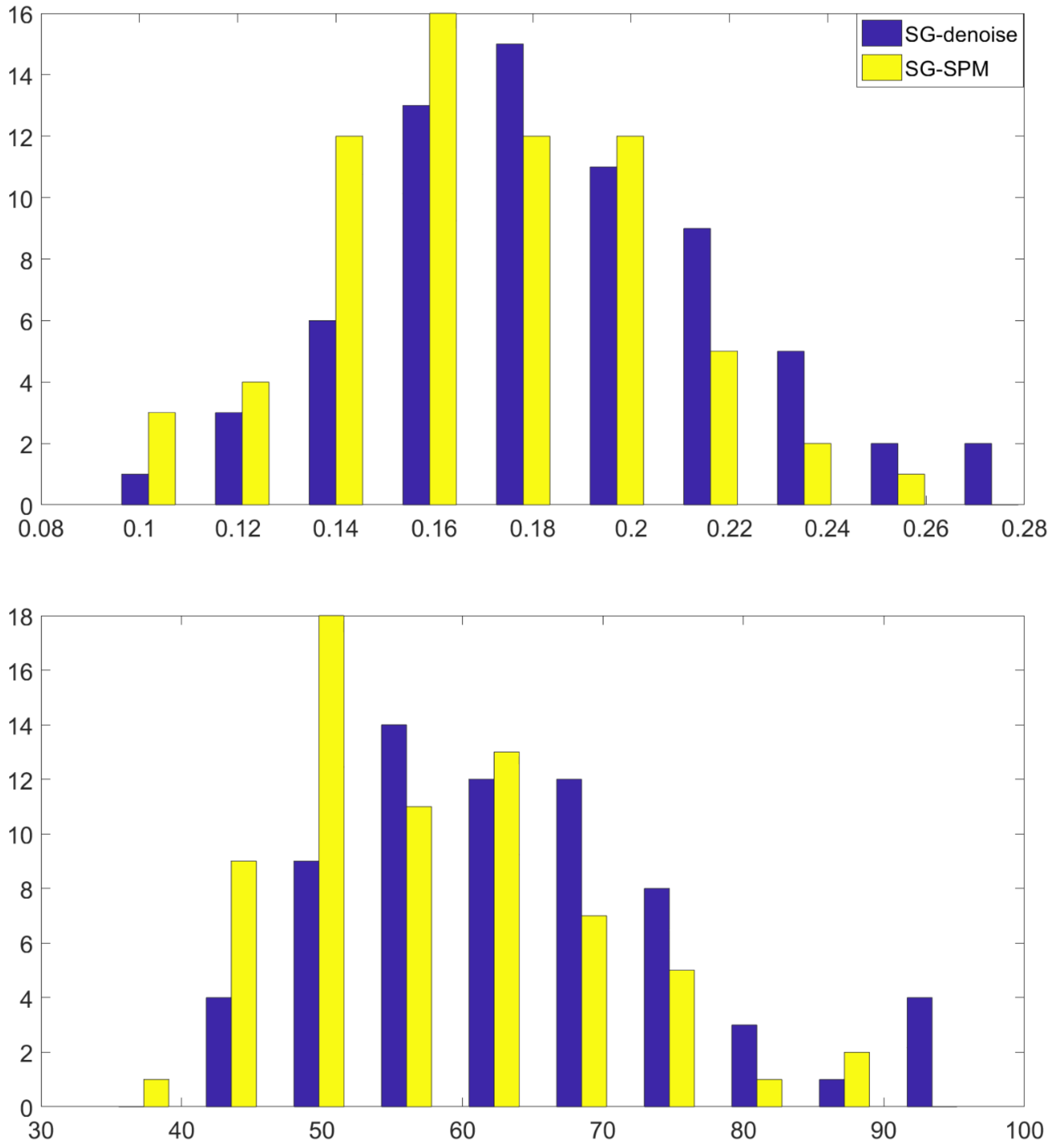

Figure S9: The reliability improvement of the SG pipeline compared to the denoised and SPM pipelines was assessed by subtracting reliability estimates per subject for each pipeline comparison and averaging the results pe subject. The SG pipeline included 12 nuisance regressors, detrending with an SG (69/6) filter, and cleaning with a (15/8) filter. The denoised pipeline also used 12 nuisance regressors, while the SPM pipeline included 12 nuisance regressors and a detrend filter (128 seconds). A histogram was generated to visually represent both the reliability improvements shown as correlation differences (top), as well as the relative improvements displayed in percentages (bottom). No negative values were observed, indicating that all individuals benefited from the SG pipeline.

S10 Figure. Across pipeline comparisons

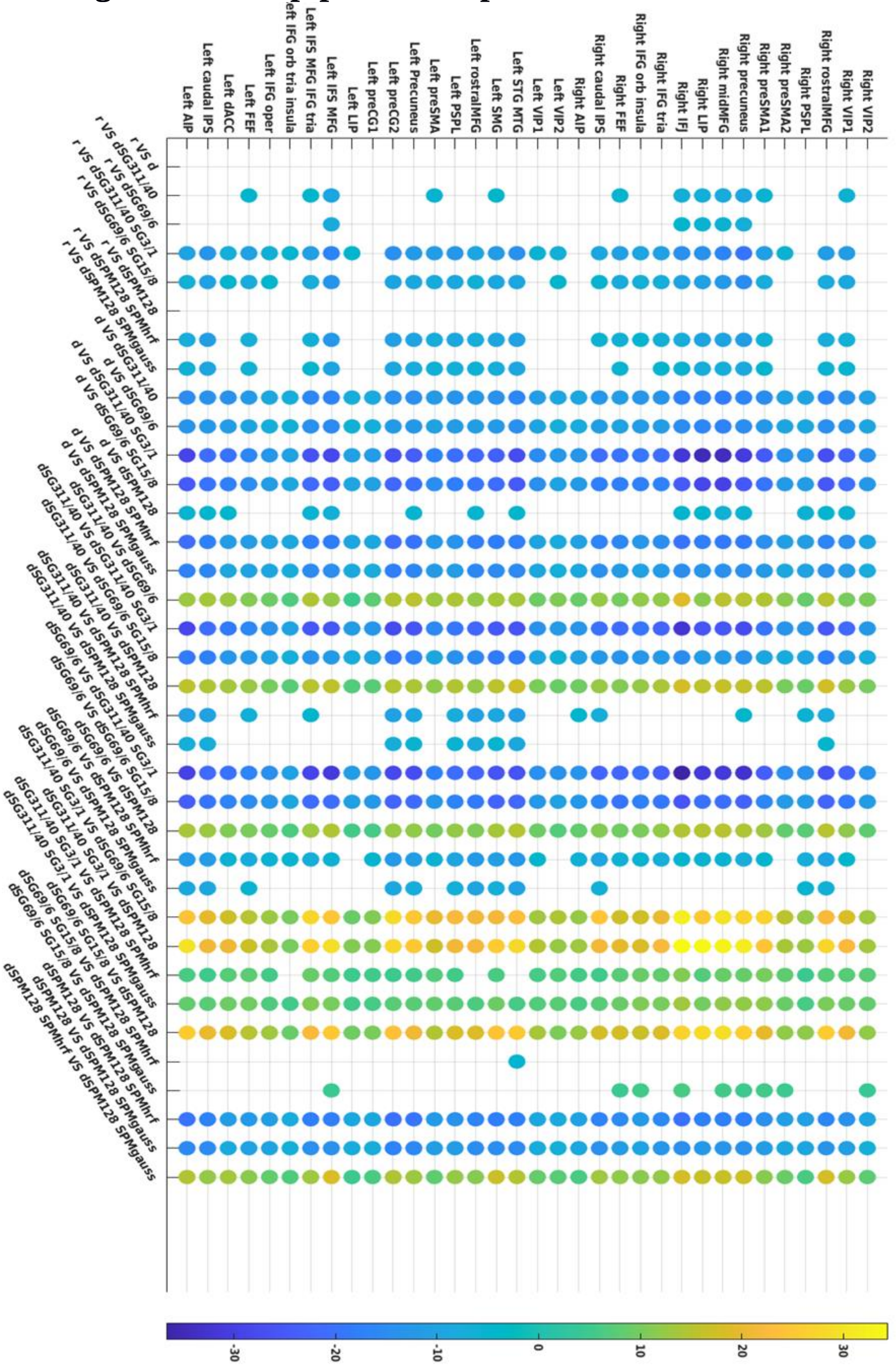

Figure S10: This figure illustrates the statistical differences in test-retest reliability among the distinct pipelines for each region of interest. The horizontal axis represents the regions of interest, while the vertical axis indicates which pipelines were compared against each other. The abbreviation "VS." stands for "versus," "r" refers to "raw," and "d" denotes "denoised." The detrending and cleaning filters are labeled according to the nomenclature described in the main text. The color bar at the bottom indicates the significance of the results and is expressed in t values. Please note that the t value is displayed in blue when the pipeline in the first position exhibits less reliability than that in the second position. Conversely, it appears in warm yellow when the second position shows lower reliability than the first. Regions that did not survive Bonferroni correction are not displayed, calculated as 0.05 divided by the product of the number of pipeline comparisons and the number of regions.

S11 Figure. Connectomes

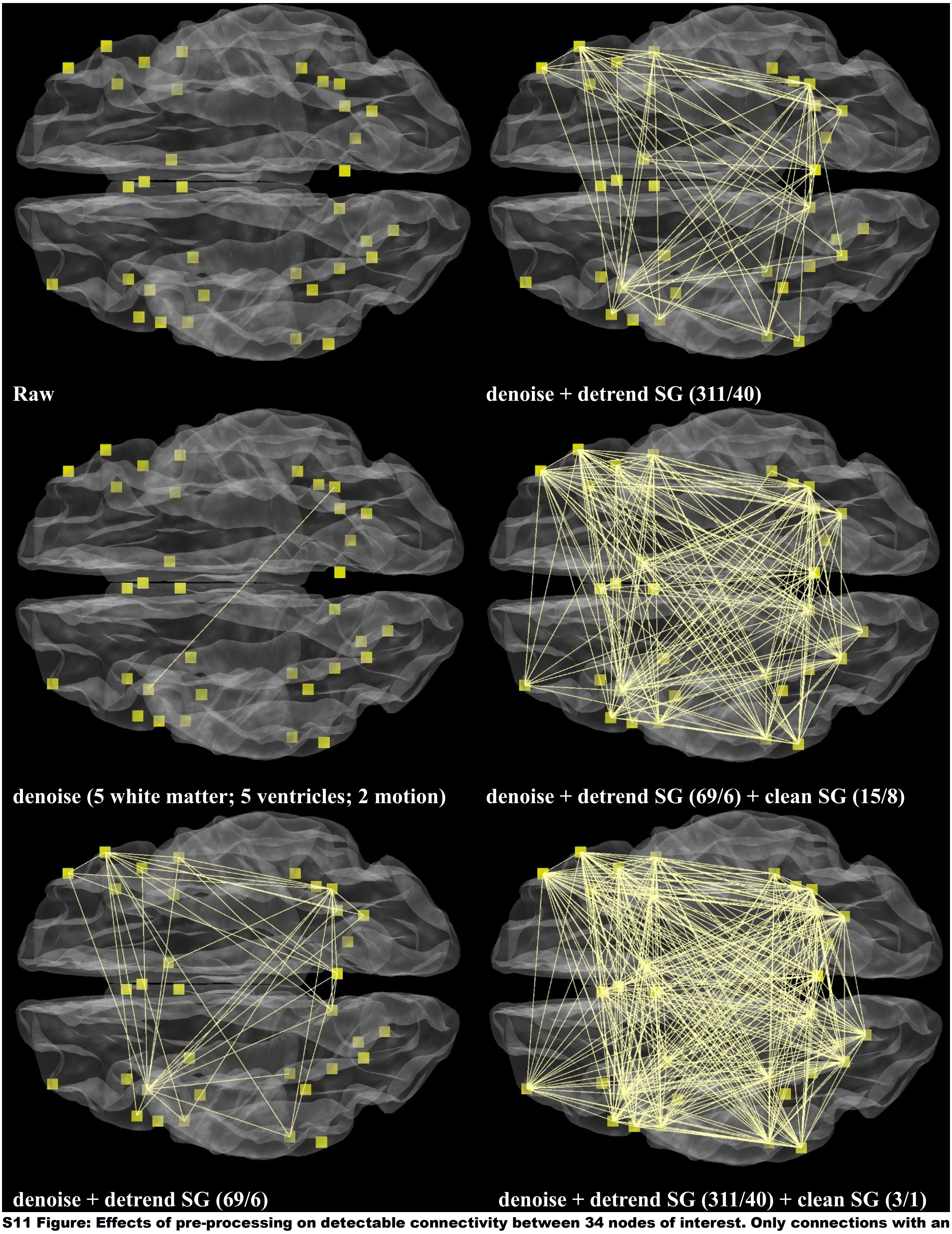

S11 Figure: Effects of pre-processing on detectable connectivity between 34 nodes of interest. Only connections with an average detectable connectivity of r>0.4 are shown. An average detectable connectivity of r>0.4 implies that average within-subject time-course reliability of the underlying nodes is at least fair (i.e. r>0.4).

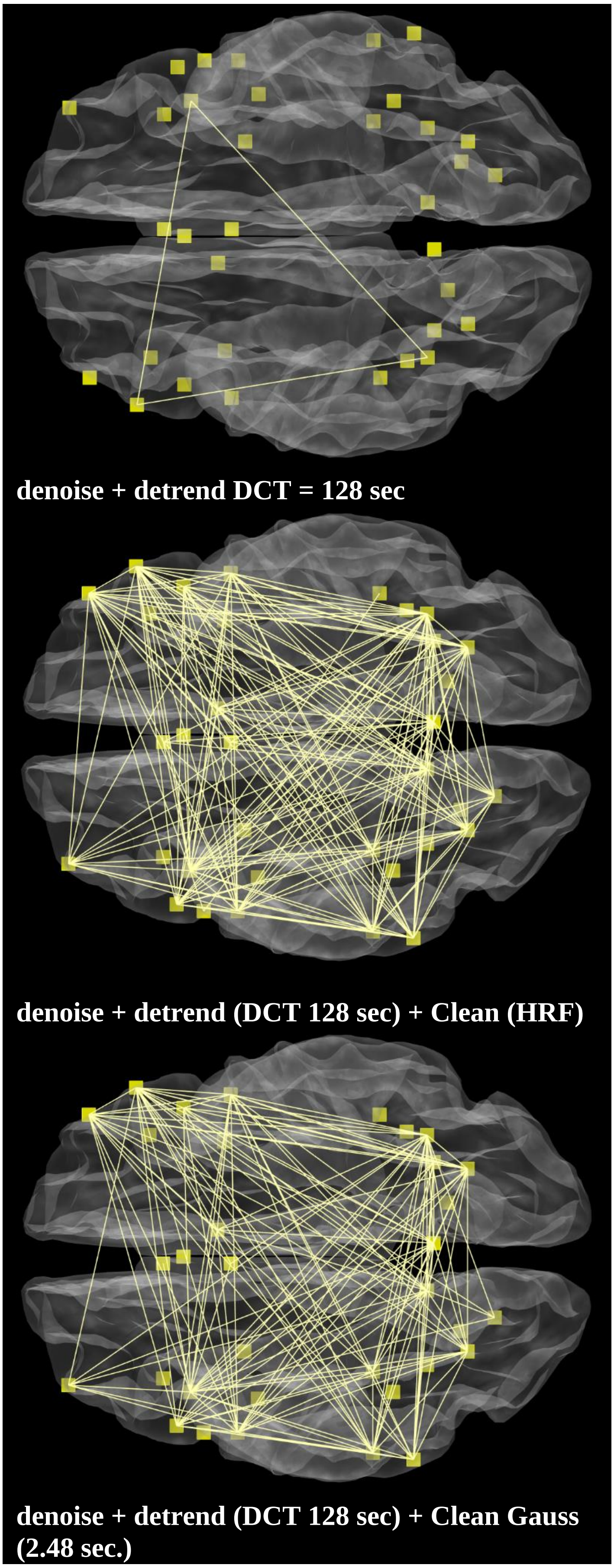

S11 Figure: Effects of pre-processing on detectable connectivity between 34 nodes of interest. Only connections with an average detectable connectivity of r>0.4 are shown. An average detectable connectivity of r>0.4 implies that average within-subject time-course reliability of the underlying nodes is at least fair (i.e. r>0.4).

S12 Figure. FFT analysis

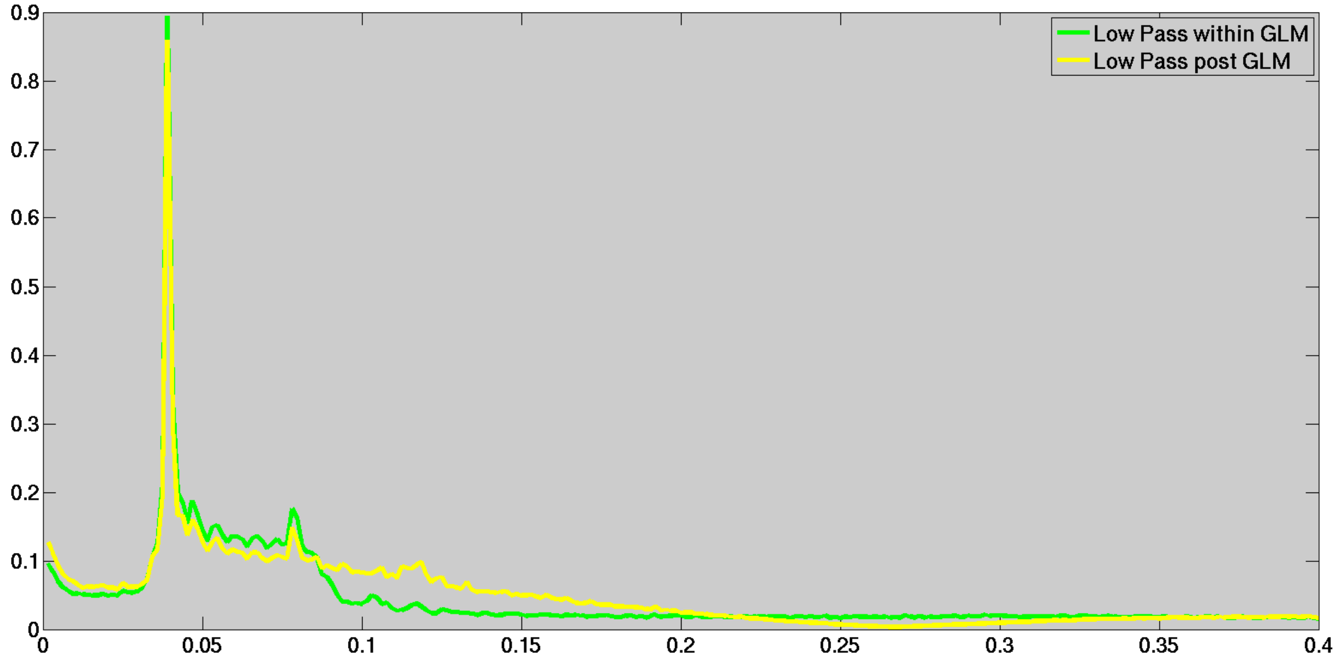

S12 Figure: This figure shows the effect of preprocessing on the power spectra of working state data. The grand mean FFTs were obtained by averaging 4556 FFTs that were obtained from 34 nodes*2runs*67subjects. The yellow line reflects the power spectra of the optimal filter set that was achieved by developing the optimal cleaning filter after time courses were detrended and denoised within a GLM frame work. SG3/1(glm(Denoised+Detrend SG (311/40)) The green line reflects the power spectra of the optimal cleaning filter set that was achieved by developing the optimal cleaning filter in concert with detrending and denoising within a GLM frame work. glm(Denoised+Detrend SG (311/40)+Filtered SG (105/35)).

S13 Figure. Simulation

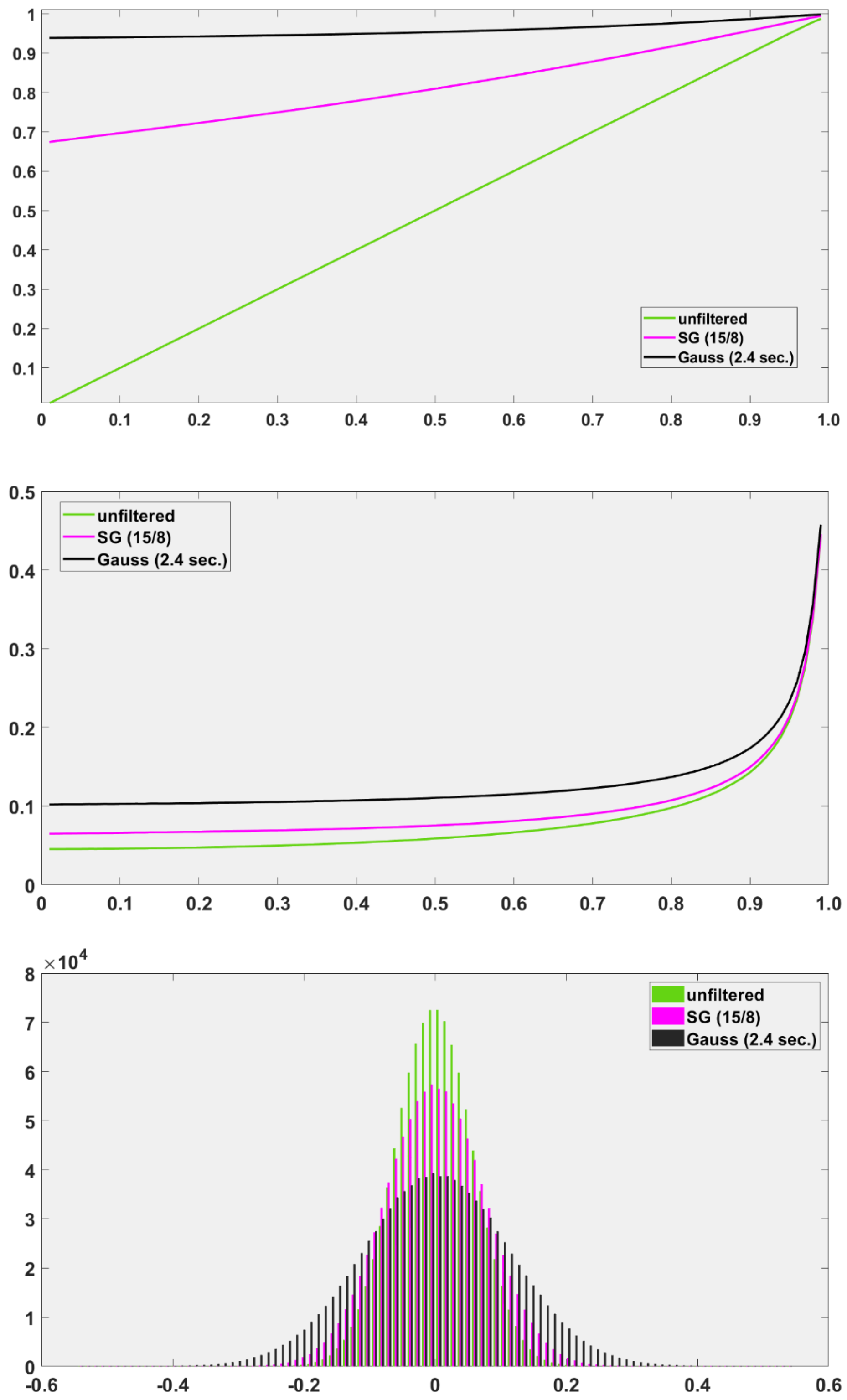

S13 Figure: Reports the effect of an SG (15/8) and Gaussian filter (2.4 sec.) on the autocorrelation behavior of simulated time courses, standard deviations of time course reproducibility correlations, and distributions of time course reproducibility correlations. Top: the horizontal axis displays the autocorrelation of simulated time courses before filtering the vertical axis displays the average autocorrelation after filtering. Middle: the horizontal axis displays the autocorrelation of the two simulated time courses that were entered into the time course reproducibility estimation, the vertical axis displays the standard deviation of the time course reproducibility correlations. Note that for top and middle displays statistics were estimated using fisher z transform/inverse transform. Bottom: fishers z-transformed distributions of time course reproducibility correlations before and after filtering based on time courses with a lag 1 autocorrelation of 0.51.

S1 Table. Autocorrelation of predictor time course

	Auto correlation of predictor time course of test run								
	Raw	Denoised	Denoised Detrended SG (311/40)	Denoised Detrended SG (69/6)	Denoised Detrended SG (311/40) Filtered SG (3/1)	Denoised Detrended SG (69/6) Filtered SG (15/8)	Denoised Detrended SPM (128 s.)	Denoised Detrended SPM (128s) Filtered SPM (HRF)	Denoised Detrended SPM (128s) Filtered SPM Gauss (2.48 s.)
Lag 1	0.80	0.79	0.82	0.82	0.89	0.88	0.79	0.92	0.92
Lag 2	0.51	0.49	0.55	0.54	0.64	0.59	0.50	0.70	0.71
Lag 3	0.21	0.20	0.26	0.25	0.32	0.26	0.21	0.41	0.42
Lag 4	-0.05	-0.03	0.00	0.00	0.02	-0.01	-0.03	0.08	0.09
	Auto correlation of predictor time course of retest run								
	Raw	Denoised	Denoised Detrended SG (311/40)	Denoised Detrended SG (69/6)	Denoised Detrended SG (311/40) Filtered SG (3/1)	Denoised Detrended SG (69/6) Filtered SG (15/8)	Denoised Detrended SPM (128 s.)	Denoised Detrended SPM (128s) Filtered SPM (HRF)	Denoised Detrended SPM (128s) Filtered SPM Gauss (2.48 s.)
Lag 1	0.78	0.78	0.82	0.81	0.89	0.88	0.79	0.91	0.92
Lag 2	0.49	0.49	0.55	0.54	0.63	0.59	0.49	0.70	0.71
Lag 3	0.20	0.20	0.25	0.24	0.31	0.25	0.20	0.40	0.41
Lag 4	-0.07	-0.05	-0.02	-0.01	0.01	-0.03	-0.05	0.07	0.08

S1 Table: This table reports the average auto correlation structure (lag 1– 4) of predicted time courses that were based on event related averages of the distinct preprocessing methods for the test and retest run. The autocorrelations were obtained by averaging the 2278 autocorrelation obtained from 34 paths * 67 individuals.

S2 Table. Test-retest reliability per region

Region	X	Y	Z	Raw	Denoised	Denoised	Denoised	Denoised	Denoised	Denoised	Denoised	Denoised
				slice time	5 PC wm	Detrended	Detrended	Detrended SG (311/40)	Detrended SG (69/6)	Detrended	Detrended	Detrended
				Motion correction)	5 PC ventricle	SG (311/40)	SG (69/6)	Cleaned	Cleaned	SPM (128s.)	SPM (128s.)	SPM (128s.)
					2 PC motion			SG (3/1)	SG (15/8)		Cleaned	Cleaned
											SPM (HRF)	SPM(2.48s.)
											Filter	Gauss filter
Left_AIP	-34	-38	42	0.31	0.26	0.35	0.33	0.52	0.45	0.27	0.47	0.44
Left_caudal_IPS	-28	-66	36	0.28	0.25	0.34	0.32	0.50	0.44	0.26	0.46	0.43
Left_dACC	-2	24	38	0.26	0.21	0.30	0.28	0.44	0.38	0.22	0.36	0.33
Left_FEF	-28	0	58	0.19	0.20	0.27	0.25	0.40	0.34	0.20	0.33	0.31
Left_IFG_oper	-52	12	22	0.20	0.19	0.27	0.25	0.39	0.32	0.20	0.32	0.30
Left_IFG_orb_tria_insula	-36	24	2	0.11	0.10	0.16	0.15	0.25	0.21	0.12	0.21	0.19
Left_IFS_MFG_IFG_tria	-50	20	28	0.31	0.27	0.39	0.37	0.53	0.46	0.29	0.45	0.41
Left_IFS_MFG	-40	16	28	0.41	0.42	0.55	0.53	0.65	0.60	0.44	0.59	0.55
Left_LIP	-40	-44	50	0.17	0.12	0.19	0.18	0.28	0.24	0.14	0.23	0.21
Left_preCG1	-42	-4	42	0.19	0.13	0.21	0.19	0.31	0.26	0.15	0.24	0.22
Left_preCG2	-52	2	42	0.33	0.29	0.39	0.37	0.56	0.48	0.30	0.51	0.48
Left_Precuneus	-10	-54	48	0.38	0.34	0.45	0.42	0.59	0.53	0.36	0.55	0.51
Left_preSMA	-2	4	62	0.24	0.22	0.32	0.30	0.44	0.38	0.23	0.37	0.35
Left_PSPL	-18	-74	42	0.25	0.21	0.29	0.27	0.44	0.37	0.22	0.39	0.36
Left_rostralMFG	-38	52	10	0.22	0.20	0.28	0.26	0.43	0.37	0.21	0.40	0.37
Left_SMG	-58	-38	32	0.32	0.31	0.42	0.39	0.57	0.50	0.33	0.52	0.48
Left_STG_MTG	-60	-50	10	0.32	0.26	0.34	0.32	0.51	0.44	0.27	0.48	0.45
Left_VIP1	-22	-64	54	0.25	0.17	0.26	0.24	0.38	0.31	0.19	0.31	0.29
Left_VIP2	-32	-54	47	0.12	0.12	0.21	0.18	0.31	0.24	0.13	0.22	0.20
Right_AIP	42	-40	50	0.27	0.18	0.26	0.24	0.37	0.31	0.20	0.31	0.29
Right_caudal_IPS	26	-66	38	0.36	0.29	0.39	0.36	0.53	0.46	0.30	0.47	0.44
Right_FEF	34	6	56	0.19	0.22	0.32	0.30	0.45	0.39	0.23	0.37	0.33
Right_IFG_orb_insula	36	28	2	0.24	0.20	0.30	0.28	0.43	0.37	0.22	0.34	0.31
Right_IFG_tria	44	18	4	0.32	0.29	0.39	0.36	0.52	0.45	0.30	0.44	0.41
Right_IFJ	48	4	38	0.35	0.32	0.46	0.43	0.58	0.51	0.34	0.48	0.45
Right_LIP	36	-54	48	0.41	0.42	0.53	0.51	0.64	0.58	0.43	0.58	0.54
Right_midMFG	50	32	34	0.42	0.39	0.52	0.50	0.64	0.57	0.41	0.56	0.52
Right_precuneus	4	-56	54	0.37	0.35	0.47	0.44	0.59	0.53	0.37	0.52	0.48
Right_preSMA1	8	8	68	0.31	0.29	0.41	0.38	0.52	0.45	0.30	0.43	0.40
Right_preSMA2	0	18	50	0.25	0.20	0.30	0.28	0.40	0.34	0.21	0.31	0.29
Right_PSPL	16	-60	54	0.22	0.14	0.21	0.20	0.33	0.27	0.16	0.28	0.26
Right_rostralMFG	42	46	26	0.36	0.30	0.40	0.38	0.56	0.49	0.31	0.50	0.46
Right_VIP1	28	-56	60	0.30	0.27	0.40	0.38	0.53	0.46	0.30	0.44	0.41
Right_VIP2	37	-48	35	0.22	0.16	0.27	0.24	0.37	0.31	0.18	0.28	0.25

S3 Table. SD of test-retest reliability per region

Region	X	Y	Z	Raw	Denoised	Denoised	Denoised	Denoised	Denoised	Denoised	Denoised	Denoised
				slice time	5 PC wm	Detrended	Detrended	Detrended SG (311/40)	Detrended SG (69/6)	Detrended	Detrended	Detrended
				Motion correction)	5 PC ventricle	SG (311/40)	SG (69/6)	Cleaned	Cleaned	SPM (128s.)	SPM (128s.)	SPM (128s.)
					2 PC motion			SG (3/1)	SG (15/8)		Cleaned	Cleaned
											SPM (HRF)	SPM(2.48s.)
											Filter	Gauss filter
Left_AIP	-34	-38	42	0.21	0.14	0.17	0.16	0.21	0.19	0.14	0.21	0.21
Left_caudal_IPS	-28	-66	36	0.18	0.13	0.16	0.16	0.22	0.19	0.14	0.22	0.21
Left_dACC	-2	24	38	0.25	0.13	0.14	0.14	0.19	0.18	0.13	0.22	0.22
Left_FEF	-28	0	58	0.15	0.12	0.15	0.14	0.20	0.18	0.12	0.20	0.19
Left_IFG_oper	-52	12	22	0.19	0.14	0.18	0.17	0.24	0.20	0.14	0.23	0.22
Left_IFG_orb_tria_insula	-36	24	2	0.17	0.12	0.14	0.13	0.21	0.18	0.11	0.20	0.19
Left_IFS_MFG_IFG_tria	-50	20	28	0.22	0.14	0.17	0.17	0.20	0.19	0.14	0.21	0.20
Left_IFS_MFG	-40	16	28	0.24	0.17	0.18	0.18	0.20	0.19	0.17	0.23	0.22
Left_LIP	-40	-44	50	0.20	0.11	0.14	0.13	0.19	0.16	0.11	0.18	0.18
Left_preCG1	-42	-4	42	0.23	0.10	0.13	0.12	0.19	0.16	0.10	0.18	0.17
Left_preCG2	-52	2	42	0.20	0.14	0.18	0.17	0.21	0.20	0.14	0.22	0.21
Left_Precuneus	-10	-54	48	0.19	0.16	0.20	0.20	0.24	0.22	0.16	0.23	0.22
Left_preSMA	-2	4	62	0.18	0.16	0.19	0.18	0.25	0.22	0.16	0.24	0.23
Left_PSPL	-18	-74	42	0.15	0.13	0.15	0.14	0.21	0.19	0.13	0.23	0.22
Left_rostralMFG	-38	52	10	0.22	0.12	0.13	0.13	0.18	0.17	0.11	0.20	0.19
Left_SMG	-58	-38	32	0.24	0.16	0.18	0.17	0.22	0.20	0.16	0.24	0.23
Left_STG_MTG	-60	-50	10	0.18	0.13	0.15	0.14	0.19	0.18	0.14	0.23	0.22
Left_VIP1	-22	-64	54	0.20	0.14	0.16	0.15	0.23	0.19	0.14	0.24	0.23
Left_VIP2	-32	-54	47	0.23	0.11	0.13	0.13	0.19	0.17	0.10	0.17	0.16
Right_AIP	42	-40	50	0.24	0.13	0.16	0.15	0.22	0.19	0.13	0.20	0.20
Right_caudal_IPS	26	-66	38	0.23	0.14	0.17	0.17	0.21	0.20	0.15	0.22	0.21
Right_FEF	34	6	56	0.26	0.12	0.15	0.14	0.19	0.17	0.12	0.17	0.16
Right_IFG_orb_insula	36	28	2	0.19	0.12	0.16	0.15	0.20	0.18	0.12	0.20	0.19
Right_IFG_tria	44	18	4	0.20	0.17	0.20	0.19	0.24	0.23	0.18	0.25	0.24
Right_IFJ	48	4	38	0.18	0.13	0.15	0.14	0.16	0.15	0.13	0.17	0.17
Right_LIP	36	-54	48	0.25	0.16	0.18	0.18	0.19	0.19	0.16	0.21	0.20
Right_midMFG	50	32	34	0.21	0.16	0.17	0.16	0.19	0.18	0.16	0.22	0.21
Right_precuneus	4	-56	54	0.20	0.17	0.19	0.19	0.21	0.20	0.17	0.22	0.22
Right_preSMA1	8	8	68	0.20	0.15	0.16	0.16	0.20	0.19	0.14	0.21	0.20
Right_preSMA2	0	18	50	0.23	0.13	0.18	0.17	0.23	0.20	0.13	0.20	0.19
Right_PSPL	16	-60	54	0.25	0.12	0.15	0.15	0.22	0.19	0.13	0.21	0.20
Right_rostralMFG	42	46	26	0.19	0.15	0.17	0.17	0.21	0.20	0.16	0.24	0.23
Right_VIP1	28	-56	60	0.19	0.15	0.18	0.18	0.21	0.19	0.16	0.23	0.22
Right_VIP2	37	-48	35	0.29	0.11	0.15	0.14	0.21	0.18	0.12	0.19	0.19

S4 Table. Autocorrelation of integrated cleaning filters

	observed auto correlation test run				observed auto correlation re test run			
	lag 1	lag 2	lag 3	lag 4	lag 1	lag 2	lag 3	lag 4
Denoised<br>Detrend SG (311/40)<br>Filtered SG (105/35)	0.87	0.66	0.39	0.09	0.87	0.66	0.37	0.06
Denoised<br>Detrend SPM (128 s.)<br>Filter SPM (HRF)	0.88	0.73	0.53	0.32	0.88	0.74	0.55	0.34
Denoised<br>Detrend SPM (128 s.)<br>Filter SPM Gaussian (2.48 s.)	0.84	0.73	0.58	0.40	0.85	0.74	0.61	0.44
	predicted auto correlation of event related average re test run				predicted auto correlation of event related average test run			
	lag 1	lag 2	lag 3	lag 4	lag 1	lag 2	lag 3	lag 4
Denoised	0.78	0.49	0.20	-0.05	0.79	0.49	0.20	-0.03

S4 Table: This table reports the effects of filters on the autocorrelation structure of time courses. In this case noise, detrend filters and cleaning filters were all regressed out simultaneously.

S5 Table. Reliability of integrated cleaning filters

	Entire time courses		
Within subject within path Reliability	Denoised Detrend SG (311/40) Filtered SG (105/35)	Denoised Detrended SPM (128s.) Filtered SPM (HRF)	Denoised Detrended SPM (128s.) Filtered SPM (2.48s.)
Grand mean Connectivity estimate	0.66	0.60	0.58
Grand mean reliability estimate	0.53	0.39	0.35

S5 Table: This table reports test-retest reliability and connectivity statistics of working state data on the within subject level as a function of preprocessing method. Data were estimated from a connectome that consisted of 34 nodes obtained from 67 subjects with a test-retest design. However, in contrast to table 2, noise regressors, detrend and cleaning filters were all regressed out simultaneously.

References

1. Hallquist MN, Hwang K, Luna B. The nuisance of nuisance regression: spectral misspecification in a common approach to resting-state fMRI preprocessing reintroduces noise and obscures functional connectivity. Neuroimage. 2013; 82:208–25. Epub 2013/06/06. doi: 10.1016/j.neuroimage.2013.05.116 PMID: 23747457.
